# Supplementary material for: A warm-up strategy with or without voluntary contraction on athletic performance, lower-leg temperature, and blood lactate concentration
Source: PLoS One. 2024 Jan 11;19(1):e0295537. doi: 10.1371/journal.pone.0295537 (PMC10783781; doi:10.1371/journal.pone.0295537)
Supplement: Attachment — Submitted filename: Responses to Reviewers.docx [file pone.0295537.s002.docx]

Dear Authors,

one expert in the field reviewed your manuscript, retrieving several majors you should consider during the revision process.

Please submit your revised manuscript by Aug 18 2023 11:59PM. If you will need more time than this to complete your revisions, please reply to this message or contact the journal office at [plosone@plos.org](mailto:plosone@plos.org). When you're ready to submit your revision, log on to <https://www.editorialmanager.com/pone/> and select the 'Submissions Needing Revision' folder to locate your manuscript file.

Please include the following items when submitting your revised manuscript:

- A rebuttal letter that responds to each point raised by the academic editor and reviewer(s). You should upload this letter as a separate file labeled 'Response to Reviewers'.
- A marked-up copy of your manuscript that highlights changes made to the original version. You should upload this as a separate file labeled 'Revised Manuscript with Track Changes'.
- An unmarked version of your revised paper without tracked changes. You should upload this as a separate file labeled 'Manuscript'.

If you would like to make changes to your financial disclosure, please include your updated statement in your cover letter. Guidelines for resubmitting your figure files are available below the reviewer comments at the end of this letter.

If applicable, we recommend that you deposit your laboratory protocols in [protocols.io](http://protocols.io/) to enhance the reproducibility of your results. Protocols.io assigns your protocol its own identifier (DOI) so that it can be cited independently in the future. For instructions see: <https://journals.plos.org/plosone/s/submission-guidelines#loc-laboratory-protocols>. Additionally, PLOS ONE offers an option for publishing peer-reviewed Lab Protocol articles, which describe protocols hosted on [protocols.io](http://protocols.io/). Read more information on sharing protocols at <https://plos.org/protocols?utm_medium=editorial-email&utm_source=authorletters&utm_campaign=protocols>.

We look forward to receiving your revised manuscript.

Kind regards,

Emiliano Cè
Academic Editor
PLOS ONE

Journal requirements:

When submitting your revision, we need you to address these additional requirements.

1. Please ensure that your manuscript meets PLOS ONE's style requirements, including those for file naming. The PLOS ONE style templates can be found at

<https://journals.plos.org/plosone/s/file?id=wjVg/PLOSOne_formatting_sample_main_body.pdf> and

<https://journals.plos.org/plosone/s/file?id=ba62/PLOSOne_formatting_sample_title_authors_affiliations.pdf>

Our manuscript meets the requirements.

2. We note that you have stated that you will provide repository information for your data at acceptance. Should your manuscript be accepted for publication, we will hold it until you provide the relevant accession numbers or DOIs necessary to access your data. If you wish to make changes to your Data Availability statement, please describe these changes in your cover letter and we will update your Data Availability statement to reflect the information you provide.

We will provide the data once our paper is accepted.

3. Please include your full ethics statement in the ‘Methods’ section of your manuscript file. In your statement, please include the full name of the IRB or ethics committee who approved or waived your study, as well as whether or not you obtained informed written or verbal consent. If consent was waived for your study, please include this information in your statement as well.

The full name of the IRB committee has been added (Ln 80-81). We have obtained written consent, which has been stated (Ln 80-81).

4. Please include captions for your Supporting Information tables at the end of your manuscript, and update any in-text citations to match accordingly. Please see our Supporting Information guidelines for more information: <http://journals.plos.org/plosone/s/supporting-information>.

We have followed the guidelines and revised captions accordingly.

[Note: HTML markup is below. Please do not edit.]

Reviewers' comments:

Reviewer's Responses to Questions

**Comments to the Author**

1. Is the manuscript technically sound, and do the data support the conclusions?
The manuscript must describe a technically sound piece of scientific research with data that supports the conclusions. Experiments must have been conducted rigorously, with appropriate controls, replication, and sample sizes. The conclusions must be drawn appropriately based on the data presented.

Reviewer #1: No

2. Has the statistical analysis been performed appropriately and rigorously?

Reviewer #1: Yes

3. Have the authors made all data underlying the findings in their manuscript fully available?
The [PLOS Data policy](http://www.plosone.org/static/policies.action#sharing) requires authors to make all data underlying the findings described in their manuscript fully available without restriction, with rare exception (please refer to the Data Availability Statement in the manuscript PDF file). The data should be provided as part of the manuscript or its supporting information, or deposited to a public repository. For example, in addition to summary statistics, the data points behind means, medians and variance measures should be available. If there are restrictions on publicly sharing data—e.g. participant privacy or use of data from a third party—those must be specified.

Reviewer #1: No

4. Is the manuscript presented in an intelligible fashion and written in standard English?
PLOS ONE does not copyedit accepted manuscripts, so the language in submitted articles must be clear, correct, and unambiguous. Any typographical or grammatical errors should be corrected at revision, so please note any specific errors here.

Reviewer #1: No

5. Review Comments to the Author
Please use the space provided to explain your answers to the questions above. You may also include additional comments for the author, including concerns about dual publication, research ethics, or publication ethics. (Please upload your review as an attachment if it exceeds 20,000 characters)

Reviewer #1: I would like to congratulate the authors for the effort and time spent conducting this study. This is an interesting study under the scope of the journal, and that could provide some practical applications for coaches. However, in my opinion, there are some major concerns regarding the methodology and evaluations that can compromise results. This is explained in specific comments and should be clarified before further revision.

We would like to thank the editor and reviewer for critically reviewing our manuscript and providing valuable comments. Below, you can find a list of changes regarding your comments and our responses (highlighted in yellow). The changes in the revised manuscript were also highlighted in yellow. Thanks again for your valuable comments.

Specific comments:
- The authors should clarify the use of some specific words. For example, what is the difference between voluntary and involuntary? Are the authors talking about active and passive warm-ups? Both exercises were voluntary. The main difference between them was performing physical exercise. This concept o voluntary or involuntary should be rethanked or clarified throughout the manuscript.

We agree with the reviewer that the terms *voluntary* and *involuntary* can be confused. We have defined active and passive warm-ups with appropriate references (Ln 10-14) and have renamed them throughout the manuscript.

- English writing should be revised throughout the manuscript

The revised manuscript has been proofread.

- I think I understand the sentence, “Non-thermal effect from the warm-up activity may not be necessary for sprinting and jumping performance in recreationally active males.”. however, there are two negative sentences, and this should be rewritten.

This sentence has been revised (last sentence in the abstract).

- The hypothesis should be rewritten to replace the “similar” word. The hypothesis should be “different” or “not different”. Being not different is not the same as “similar”.

This has been revised, as the reviewer requested (Ln 52).

- The laboratory temperature is higher than usual for this kind of experiment (~26ºC). Could this have influenced the results? Please, clarify and, if needed, include it in the discussion.

This has been added as a limitation as the reviewer requested (Ln 321-3).

- What were the level and/or experience of participants? Warm-up effects depend on the level and experience of participants, and this should be presented.

As the reviewer requested, the inclusion criterion on the activity level in our subjects was added in the subject description paragraph (Ln 74-6). The average exercise duration was also added in the limitation paragraph (Ln 318-21).

- Each participant performed a maximal vertical jump at baseline. This was performed before the voluntary and involuntary warm-up. So, there was physical exercise, together with the involuntary warm-up, in some participants (those randomly assigned to this condition on their first visit). Could this activation influence results? Please, discuss or clarify.

This has been added as a limitation, as the reviewer requested (Ln 330-2).

- Vertical jump was determined by vertec equipment, and this has strong limitations. Other equipment provides a more reliable measurement of the vertical jump. Moreover, in this kind of jump test, the participants' jump can be affected by several factors that should be considered, such as shoulder range of motion, arm swings, dominant and non-dominant hand, and bending. So, the authors should support their choice with a validation study of this procedure.

We have added a statement regarding the validation and reliability of the jump-reach test in the similar subject population (recreationally active college students: Ln 86-90). Our subjects were all right-dominant, which has been updated in the methods section (Ln 92). Although it is a valid and reliable test tool, we agree with the reviewer’s point here that the jump-reach method requires body coordination. We have added this as a potential limitation (332-7). Our laboratory has previously used this device to collect vertical jump data on both athletic and general populations and has published several papers using said data (papers that are not cited here due to blind review): for three of those recently published papers, high reliabilities (ICC of 0.95-0.98; 0.94-0.97; 0.94-0.98) were reported. Although the current study design did not allow us to calculate ICC values (no baseline values were collected), we believe that the measurement consistency issue was minimal.

- Please, be specific to the time of collecting data (blood lactate and temperatures) after warm-up and after the main exercise to allow replication. And, if appropriate, what was used for analysis.

To clarify the measurement time points, we have modified Figure 1 and the related captions. The analysis processes for blood lactate levels and muscle temperature were described in the method section (Ln 109-126).

- The performance measure was recorded by using an intermittent exercise of 23 min. However, during this exercise, the intensity was self-determined, requiring a maximal intensity only in some parts of the test. This is kind of hard to understand if this should be a reliable measure of performance for several reasons. For example, how should we know that each one performed at 25% or 75% in both conditions, and some kind of saving energy exists and then contributes to the maximization of 100% intensity effort? Then, the authors report that to examine performance after two warm-up protocols, 20m sprint time and maximal vertical jump height at each set of intermittent exercises were recorded. So, as far as I understand, vertical jump and running at submaximal and maximal were performed and analysed. I am not sure about the validity of these measurements because of being submaximal and without any control (self-determined), and I am not sure about the influence of the effort management of each participant and whether each previous exercise will compromise any difference from previous warm-up conditions on subsequent exercise. This is a major issue of the study and should be clarified and discussed by the authors.

The reviewer has a valid point here. Originally (Loughborough Intermittent Shuttle Test, Nicholas, 2000), the intensities of various jumping and running movements were controlled by % VO2max, which was guided by audio feedback. This method has been updated to include the 80% maximal vertical jumps (Welsh, 2002) and was modified again by adding the 80% and 100% maximal vertical jumps (Kim, 2016). From our pilot work, we found that the use of self-determined speeds alongside prescribed cutoff times (Ln 146-9: the 80% and 100% maximal vertical jumps were completed within 40 and 90 sec, respectively) assisted subjects to maintain the running intensity during the intermittent exercises. For example, all subjects were accustomed to the running intensities after a couple of sets and had 5 to 10 sec left between sets. Therefore, we believe the intensities of intermittent exercises within and between subjects were consistent. This has been added in the limitation section (Ln 338-45).

- Nicholas et al. The Loughborough Intermittent Shuttle Test: A field test that simulates the activity pattern of soccer. Journal of Sports Science. 2000; 187: 97-104.
- Welsh et al. Carbohydrates and physical/mental performance during intermittent exercise to fatigue. Medicine & Science in Sports & Exercise. 2002; 34: 723-31.
- Kim et al. Joint cooling does not hinder athletic performance during high-intensity intermittent exercise. International Journal of Sports Medicine. 2016; 37: 641-46.

- Muscle temperature should be around 36/37º C at baseline, as presented in the literature. But the authors presented values below 33ºC. That is hypothermia, usually. All the range of values that are presented are lower than expected. There should be an explanation for these values, or please, consider removing them because it is a sign that some calibration was not correct.

We recorded the medial gastrocnemius for the muscle temperature. We believe that a body temperature around 36/37ºC is referred to as the core temperature, but this may not be the temperature in the distal muscle. Previously reported gastrocnemius muscle temperatures are listed below.

| Author  (year) | Title | Muscle temperature  (body part, technique, depth of measurement) |
| --- | --- | --- |
| Bang  (2022) | A 7-min halftime jog mitigated the reduction in sprint performance for the initial 15-min of the second half in a simulated football match | 31.1 ± 2.4°C  (gastrocnemius, noninvasive, 2.2 cm) |
| Lim  (2021) | Comparison of 4 different cooldown strategies on lower-leg temperature, blood lactate concentration, and fatigue perception after intense running | 33.1 ± 0.7°C  (gastrocnemius, noninvasive, 2.2 cm) |
| Muraoka  (2008) | Effects of muscle cooling on the stiffness of the human gastrocnemius muscle in vivo | 34.0 ± 0.8°C  (gastrocnemius, noninvasive, 2.2 cm) |
| Ichinoseki-Sekine  (2007) | Changes in muscle temperature induced by 434 MHz microwave hyperthermia | 35.0 ± 1.3°C  (vastus lateralis, noninvasive, 2 cm) |
| Garrett  (2000) | Heat distribution in the lower leg from pulsed short-wave diathermy and ultrasound treatments | 35.2 ± 1.0°C  (gastrocnemius, invasive, 3.0 cm) |
| Draper  (1995) | Rate of temperature decay in human muscle following 3 MHz ultrasound: The stretching window revealed | 33.8 ± 1.3°C  (gastrocnemius, invasive, 1.2 cm) |

Our data (32.8 ± 0.7°C) are within the ranges of previously reported data that were collected using noninvasive techniques (Bang, 2022; Lim, 2021; Muraoka, 2008). While the data collected using invasive techniques (Garrett, 2000; Draper, 1995) are considered more accurate, it seems to be that the depth of measurement is the main factor in determining the temperature. There could be a possibility of underestimation when using noninvasive methods since these noninvasive techniques are known to estimate the inner tissue temperature at a depth of 2.2 cm. We have discussed this issue as a limitation (Ln 326-31).

6. PLOS authors have the option to publish the peer review history of their article ([what does this mean?](https://journals.plos.org/plosone/s/editorial-and-peer-review-process#loc-peer-review-history)). If published, this will include your full peer review and any attached files.
If you choose “no”, your identity will remain anonymous but your review may still be made public.
**Do you want your identity to be public for this peer review?** For information about this choice, including consent withdrawal, please see our [Privacy Policy](https://www.plos.org/privacy-policy).

Reviewer #1: No

[NOTE: If reviewer comments were submitted as an attachment file, they will be attached to this email and accessible via the submission site. Please log into your account, locate the manuscript record, and check for the action link "View Attachments". If this link does not appear, there are no attachment files.]

While revising your submission, please upload your figure files to the Preflight Analysis and Conversion Engine (PACE) digital diagnostic tool, <https://pacev2.apexcovantage.com/>. PACE helps ensure that figures meet PLOS requirements. To use PACE, you must first register as a user. Registration is free. Then, login and navigate to the UPLOAD tab, where you will find detailed instructions on how to use the tool. If you encounter any issues or have any questions when using PACE, please email PLOS at [figures@plos.org](mailto:figures@plos.org). Please note that Supporting Information files do not need this step.
