# Supplementary material for: A warm-up strategy with or without voluntary contraction on athletic performance, lower-leg temperature, and blood lactate concentration
Source: PLoS One. 2024 Jan 11;19(1):e0295537. doi: 10.1371/journal.pone.0295537 (PMC10783781; doi:10.1371/journal.pone.0295537)
Supplement: Attachment — Submitted filename: Response to Reviewers_R2.docx [file pone.0295537.s003.docx]

Dear editor and reviewers:

We would like to thank you for taking time to review our manuscript and provide valuable comments. We believe that revisions made in response to your comments have clarified and strengthened the manuscript. Below is a list of changes regarding the requested revisions of the manuscript (our responses are highlighted in yellow). Changes and revisions are also highlighted in yellow in the manuscript.

Thank you again for reviewing our manuscript. We look forward to hearing from you.

Sincerely,

Authors

Dear Dr. Park,

Thank you for submitting your manuscript to PLOS ONE. After careful consideration, we feel that it has merit but does not fully meet PLOS ONE’s publication criteria as it currently stands. Therefore, we invite you to submit a revised version of the manuscript that addresses the points raised during the review process.

==============================
ACADEMIC EDITOR:

Dear Authors,

your manuscript was newly revised by one expert in the field, who still found some minor points you should consider in the revision process.

Please submit your revised manuscript by Nov 24 2023 11:59PM. If you will need more time than this to complete your revisions, please reply to this message or contact the journal office at [plosone@plos.org](mailto:plosone@plos.org). When you're ready to submit your revision, log on to <https://www.editorialmanager.com/pone/> and select the 'Submissions Needing Revision' folder to locate your manuscript file.

Please include the following items when submitting your revised manuscript:

- A rebuttal letter that responds to each point raised by the academic editor and reviewer(s). You should upload this letter as a separate file labeled 'Response to Reviewers'.
- A marked-up copy of your manuscript that highlights changes made to the original version. You should upload this as a separate file labeled 'Revised Manuscript with Track Changes'.
- An unmarked version of your revised paper without tracked changes. You should upload this as a separate file labeled 'Manuscript'.

If you would like to make changes to your financial disclosure, please include your updated statement in your cover letter. Guidelines for resubmitting your figure files are available below the reviewer comments at the end of this letter.

If applicable, we recommend that you deposit your laboratory protocols in [protocols.io](http://protocols.io/) to enhance the reproducibility of your results. Protocols.io assigns your protocol its own identifier (DOI) so that it can be cited independently in the future. For instructions see: <https://journals.plos.org/plosone/s/submission-guidelines#loc-laboratory-protocols>. Additionally, PLOS ONE offers an option for publishing peer-reviewed Lab Protocol articles, which describe protocols hosted on [protocols.io](http://protocols.io/). Read more information on sharing protocols at <https://plos.org/protocols?utm_medium=editorial-email&utm_source=authorletters&utm_campaign=protocols>.

We look forward to receiving your revised manuscript.

Kind regards,

Emiliano Cè
Academic Editor
PLOS ONE

Journal Requirements:

Please review your reference list to ensure that it is complete and correct. If you have cited papers that have been retracted, please include the rationale for doing so in the manuscript text, or remove these references and replace them with relevant current references. Any changes to the reference list should be mentioned in the rebuttal letter that accompanies your revised manuscript. If you need to cite a retracted article, indicate the article’s retracted status in the References list and also include a citation and full reference for the retraction notice.

We have carefully checked but not found any retracted articles in the reference list. As requested, we have revised the reference list (Please see yellow-highlighted);

removed the ref. #36 (Ln 352) and replaced by the ref. #7.

[Note: HTML markup is below. Please do not edit.]

Reviewers' comments:

Reviewer's Responses to Questions

**Comments to the Author**

1. If the authors have adequately addressed your comments raised in a previous round of review and you feel that this manuscript is now acceptable for publication, you may indicate that here to bypass the “Comments to the Author” section, enter your conflict of interest statement in the “Confidential to Editor” section, and submit your "Accept" recommendation.

Reviewer #1: All comments have been addressed

2. Is the manuscript technically sound, and do the data support the conclusions?

The manuscript must describe a technically sound piece of scientific research with data that supports the conclusions. Experiments must have been conducted rigorously, with appropriate controls, replication, and sample sizes. The conclusions must be drawn appropriately based on the data presented.

Reviewer #1: Yes

3. Has the statistical analysis been performed appropriately and rigorously?

Reviewer #1: Yes

4. Have the authors made all data underlying the findings in their manuscript fully available?

The [PLOS Data policy](http://www.plosone.org/static/policies.action#sharing) requires authors to make all data underlying the findings described in their manuscript fully available without restriction, with rare exception (please refer to the Data Availability Statement in the manuscript PDF file). The data should be provided as part of the manuscript or its supporting information, or deposited to a public repository. For example, in addition to summary statistics, the data points behind means, medians and variance measures should be available. If there are restrictions on publicly sharing data—e.g. participant privacy or use of data from a third party—those must be specified.

Reviewer #1: No

5. Is the manuscript presented in an intelligible fashion and written in standard English?

PLOS ONE does not copyedit accepted manuscripts, so the language in submitted articles must be clear, correct, and unambiguous. Any typographical or grammatical errors should be corrected at revision, so please note any specific errors here.

Reviewer #1: Yes

6. Review Comments to the Author

Please use the space provided to explain your answers to the questions above. You may also include additional comments for the author, including concerns about dual publication, research ethics, or publication ethics. (Please upload your review as an attachment if it exceeds 20,000 characters)

Reviewer #1: Congratulations to the authors, for their efforts in clarifying some important issues and, in my opinion, improving the quality of the manuscript. I still think that the manuscript has some important gaps that are related to the study design and procedures, as indicated in my previous review. However, the authors provided some important limitations that should be considered by the reader, when analyzing this manuscript.
However, some minor issues should be addressed, specifically:
- In the abstract, the authors continue to report that “voluntary contraction appeared to produce similar effects…” and the non-existence of difference is not the same as similar. Please, revise throughout the manuscript (i.e., discussion and conclusion).

As the reviewer requested, we have revised the related sentences in the abstract, discussion (Ln 250-251; 268-269; 302), and conclusion (Ln 346-348). We have also made additional revisions to the conclusion (please see highlighted).

- The background is clear and the introduction is supported by literature, but some evidence can be included to support it. For example, the authors reported that “Specifically, a 2% (0.7°C) core temperature increase improved sprinting and jumping performance by 6% and 4%, respectively [4]; an increase in working muscle temperature of just 1°C could affect the variation of athletic performance up to 5%. In general, muscle temperature increases rapidly during the first 3 to 5 min of warm-up and reaches a plateau after 10 to 20 min of activity [6]”. This same increase in core temperature happens in other sports, such as swimming after warm-up (e-.g., doi: 10.1519/JSC.0000000000001701), but the authors should also emphasize that this temperature can reduce immediately after ending warm-up (I.e., doi: DOI: 10.1111/j.1600-0838.2004.00349.x; doi: 10.1519/JSC.0000000000001701), and this should be discussed.

While appreciate the reviewer’s suggestion with the recommended articles here, we would like to keep the current introduction. The first paragraph (Ln 2-13) introduces the magnitudes of body temperature increase after warm-up and defines the active and passive warm-up. Since the second paragraph (Ln 14-28) talks about thermal and non-thermal effects, we thought that adding “an immediate temperature reduction after warming-up” is a different topic. Our decision was to increase readability and to link with the second paragraph. If the reviewer still thinks that “an immediate temperature reduction after warm-up” needs to be mentioned, we will be happy to add.

- I suggest that the authors divide the experimental procedures by each variable that was assessed so that can be easily understood.

As the reviewer requested, we have added appropriate subheadings.

- Please, remove data results from the discussion. New results should be included in results sessions or not discussed, and the results presented in the previous section should not be duplicated.

We have removed most data results in the discussion section. We left cited Table and Figures to provide references. We have decided to leave some of the data results for better understanding (please see highlighted). If the reviewer thinks that they still need to be removed, we will be happy to do so.

7. PLOS authors have the option to publish the peer review history of their article ([what does this mean?](https://journals.plos.org/plosone/s/editorial-and-peer-review-process#loc-peer-review-history)). If published, this will include your full peer review and any attached files.


If you choose “no”, your identity will remain anonymous but your review may still be made public.


**Do you want your identity to be public for this peer review?** For information about this choice, including consent withdrawal, please see our [Privacy Policy](https://www.plos.org/privacy-policy).

Reviewer #1: No

[NOTE: If reviewer comments were submitted as an attachment file, they will be attached to this email and accessible via the submission site. Please log into your account, locate the manuscript record, and check for the action link "View Attachments". If this link does not appear, there are no attachment files.]

While revising your submission, please upload your figure files to the Preflight Analysis and Conversion Engine (PACE) digital diagnostic tool, <https://pacev2.apexcovantage.com/>. PACE helps ensure that figures meet PLOS requirements. To use PACE, you must first register as a user. Registration is free. Then, login and navigate to the UPLOAD tab, where you will find detailed instructions on how to use the tool. If you encounter any issues or have any questions when using PACE, please email PLOS at [figures@plos.org](mailto:figures@plos.org). Please note that Supporting Information files do not need this step.
